# Preservation Strategies for Interfacial Integrity in Restorative Dentistry: A Non-Comprehensive Literature Review

**DOI:** 10.3390/jfb16020042

**Published:** 2025-01-26

**Authors:** Carmem S. Pfeifer, Fernanda S. Lucena, Fernanda M. Tsuzuki

**Affiliations:** Department of Oral Rehabilitation and Biosciences, School of Dentistry, Oregon Health & Science University, Portland, OR 97239, USA; sandesde@ohsu.edu (F.S.L.); tsuzuki@ohsu.edu (F.M.T.)

**Keywords:** restorative dentistry, resin composites, antibacterial materials

## Abstract

The preservation of interfacial integrity in esthetic dental restorations remains a critical challenge, with hybrid layer degradation being a primary factor in restoration failure. This degradation is driven by a combination of host-derived enzymatic activity, including matrix metalloproteinases (MMPs), bacterial proteases, and hydrolytic breakdown of the polymerized adhesive due to moisture exposure. This review examines the multifactorial mechanisms underlying hybrid layer degradation and presents current advancements in restorative materials aimed at counteracting these effects. Principal strategies include collagen preservation through the inhibition of enzymatic activity, the integration of antimicrobial agents to limit biofilm formation, and the use of ester-free, hydrolysis-resistant polymeric systems. Recent research highlights acrylamide-based adhesives, which exhibit enhanced resistance to acidic and enzymatic environments, as well as dual functionality in collagen stabilization. Furthermore, innovations in bioactive resins and self-healing materials present promising future directions for developing adhesives that actively contribute to long-term restoration stability. These findings underscore the importance of continuous advancements in adhesive technology to enhance the durability and clinical performance of dental restorations.

## 1. Introduction

**Figure 1:**
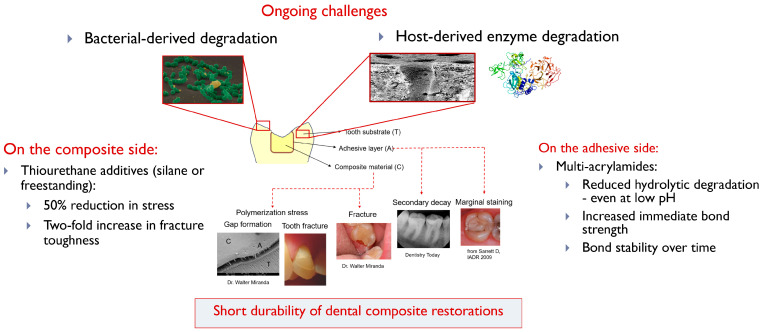
Multi-factorial process leading to the short durability of dental restorations. While strides have been made on the composite and the adhesive materials themselves (one example of improvement strategy provided for each), there are ongoing challenges related to biological factors in the oral cavity.

Interfacial integrity is crucial for the clinical longevity and effectiveness of dental restorations, as it directly impacts patient outcomes by reducing bacterial infiltration, minimizing the risk of secondary caries, and preventing premature restoration failure [1,2,3]. While the importance of the mechanical stability of the restorative material itself cannot be understated, achieving a stable, long-lasting adhesion between with the dental structure is essential for durability and reduces the frequency of re-treatments, promoting sustained oral health [4,5]. The interaction of all these factors is complex, and, thus far, the strategies to improve restoration longevity have focused on the material itself, including the strengthening of the composite with different filler technologies [6] and toughening additives [7], as well as reducing the degradation of the adhesive itself, by replacing the ester-containing methacrylates with other functionalities [8]. More recently, the focus for development of improved longevity of restorations has shifted to biological aspects that are relevant to the oral cavity (Figure 1).

A central component of interfacial integrity is the hybrid layer, a transitional zone that forms during the bonding process as adhesive resin infiltrates the partially demineralized dentin. This layer serves as a critical interface, mediating the adhesion between the restorative material and tooth structure and enhancing restoration durability. However, adhesive bonding continues to face challenges such as enzymatic degradation of the hybrid layer, moisture sensitivity, and microbial colonization, all of which compromise the durability of the adhesive interface [4,9]. Collagen degradation, a key factor in hybrid layer integrity, is primarily driven by enzymatic activity within the dentin matrix, particularly through the action of matrix metalloproteinases (MMPs). These enzymes degrade collagen fibrils within the hybrid layer, undermining the adhesive bond. Moisture exposure further exacerbates this degradation by hydrolyzing resin components, which can weaken the adhesive interface [10]. As a result, strategies to inhibit MMP activity, stabilize collagen, and develop moisture-resistant materials have emerged as critical research areas to enhance hybrid layer resilience and restoration durability [11]. In addition, biofilm formation on the restoration surface and interface also contributes to hybrid layer degradation. Biofilms consist of complex microbial communities embedded in a matrix of extracellular polymeric substances (EPS), which provide a protective barrier against the host immune response and antimicrobial agents. This localized environment fosters bacterial growth and metabolic activity, leading to continuous acid production and enzymatic degradation that weaken the components of the hybrid layer (collagen and polymerized adhesive) and surrounding tissues [12]. Strategies to inhibit bacterial colonization, either by direct bactericide or antifouling effects, have been proposed [13].

**Figure 2:**
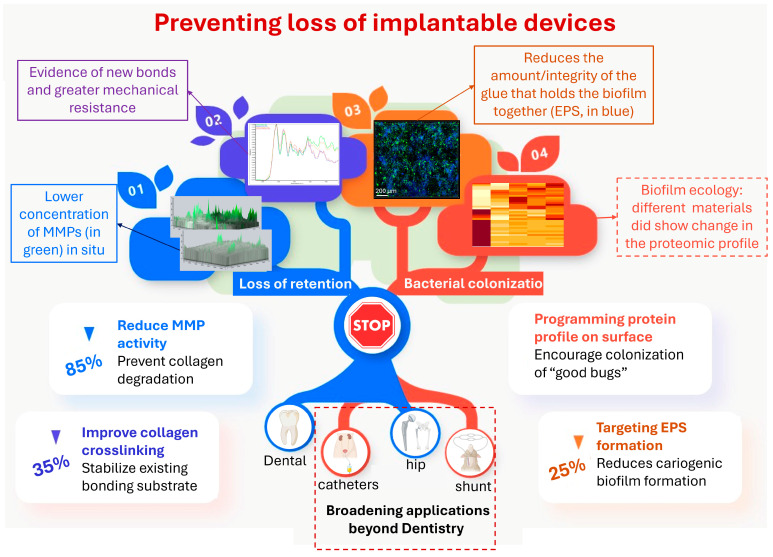
The tree diagram is rooted in preventing the loss of implantable devices, in broader biomedical applications as well as more specifically in Restorative Dentistry. The two main branches (blue or orange) indicate the two main causes for restoration loss. Each of those branches subdivide into approaches to tackle the loss of retention or the bacterial colonization. **Branch 1** shows results for in situ zymography in dentin with confocal microscopy. The bottom image in that branch shows decreased MMP activity in the sample treated with the inhibitor in comparison with the control (top image), reprinted from Ref. [14]. **Branch 2** shows infra-red spectra demonstrating the change in dentin collagen treated with acrylamide monomers, which led to complete stabilization of the bonded interface, reprinted from Ref. [14]. **Branch 3** shows a Streptococcus mutans biofilm that was treated with a GTF inhibitor, leading to decreased production of polysaccharides in the extracellular matrix (stained in blue), without killing the bacteria (stained in green), reprinted from Ref. [15]. **Branch 4** shows the proteomic profile of the acquired pellicle formed on the surface of materials with systematically increased hydrophilicity, showing enrichment of certain proteins as the hydrophilicity increases, reprinted from Ref. [16].

This review aims to provide an overview of the mechanisms contributing to hybrid layer degradation and explore innovative strategies to preserve interfacial integrity. By examining advances in antimicrobial, collagen-stabilizing, and moisture-resistant technologies over the past decade, this review highlights current progress and identifies future directions for optimizing adhesive systems in restorative dentistry (Figure 1 and Figure 2).

## 2. Overview of Hybrid Layer Formation and Structure

Dentin, as a substrate, has a complex tubular structure radiating from the pulp to the dentinoenamel junction (DEJ) [17]. This arrangement and density vary across dentin regions, with larger, more densely packed tubules near the pulp and smaller, sparser ones near the DEJ [18]. Such structural variation impacts the efficacy of adhesive systems, as regions with higher tubule density may require deeper infiltration to achieve a homogeneous hybrid layer, thus, preventing weak points that could compromise bond durability [19]. Collagen type I is the predominant organic molecule in dentin and is a robust protein with a triple-helical structure that provides high tensile strength and structural stability. The presence of non-collagenous proteins, such as proteoglycans, also plays a significant role in mineralization and collagen fibril stabilization [20]. Therefore, the properties of the dentin substrate, along with the composition of the adhesive, affect the structure of the hybrid layer, and are fundamental to determining adhesive bond durability.

The perfusion of the adhesive system within the dentinal collagen creates a bond interface that combines strength and flexibility [21]. For the systems that require acid conditioning of the dentin, typically using phosphoric acid, partial demineralization of the structure exposes a network of collagen fibers, the depth and uniformity of which determine the quality of the perfusion of the adhesive system through the collagen [22]. The acid etching procedure also results in the activation of matrix metalloproteinases, cysteins, and catepsins, which are responsible for collagen turnover and would otherwise stay dormant in the mineralized tissue [23,24]. The application of a primer leads to the enmeshing of the collagen with monomers, and increases the surface free energy, which later facilitates the interaction of the substrate with the hydrophobic bond material [22,25]. Self-etch adhesive systems, which integrate conditioning and monomer penetration, are increasingly valued for their ability to minimize the discrepancy between demineralization depth and resin penetration, promoting a more uniform and degradation-resistant interface [26,27].

## 3. Challenges in Maintaining Interfacial Integrity

The integrity of the hybrid layer in adhesive restorations is challenged by multiple degradation pathways, with hydrolytic and enzymatic processes posing primary threats [22]. Hydrolytic degradation of the adhesive polymer results in the breakdown of ester bonds in methacrylate-based adhesives in the oral environment, where moisture and pH fluctuations are constant [28,29]. This process gradually deteriorates the polymer matrix within the hybrid layer, weakening the adhesive interface and diminishing its resistance to masticatory forces over time [30]. Enzymatic degradation further undermines the stability of the hybrid layer, driven by host-derived enzymes, particularly matrix metalloproteinases (MMPs) and cysteine cathepsins, within the dentin matrix [31]. These enzymes become activated during the bonding process, especially under the acidic conditions introduced by dentin conditioning, as previously mentioned [31]. Once activated, they target exposed collagen fibrils in the hybrid layer, progressively degrading the collagen network and compromising adhesive strength. This degradation is exacerbated by salivary enzymes, which contribute to the ongoing breakdown of the collagen matrix within the hybrid layer [32].

Additionally, the resin–collagen interaction within the hybrid layer often remains inconsistent. Achieving full resin infiltration into the collagen network in clinical settings is frequently impeded by variations in substrate conditions and application techniques. Incomplete infiltration leaves voids within the hybrid layer, which become channels for moisture, bacterial penetration, and enzymatic activity, thereby accelerating degradation and shortening the functional lifespan of the adhesive bond [33,34]. In addition to these intrinsic threats, biofilm formation on adhesive surfaces presents another substantial challenge to interfacial integrity. Biofilms are complex microbial communities encased in an extracellular polymeric substance (EPS) matrix, forming a protective barrier that creates a localized acidic microenvironment. This environment promotes continuous acid and enzyme production by bacteria, accelerating both hydrolytic and enzymatic degradation of the hybrid layer. The acidic byproducts of biofilm metabolism further decrease local pH, intensifying collagen breakdown and further destabilizing the adhesive interface [35,36].

The preservation of the hybrid layer in adhesive restorations, therefore, requires an in-depth understanding of these degradation pathways, each of which directly impacts bond stability and durability. Insight into these processes—including hydrolytic and enzymatic degradation, biofilm activity, and challenges in achieving complete resin infiltration—provides essential direction for developing techniques and materials that enhance and sustain interfacial integrity in the demanding environment of the oral cavity.

### 3.1. Under-Curing the Bonding Agent

Inadequate light exposure during polymerization results in incomplete monomer conversion in the adhesive interface, leaving behind residual unpolymerized monomers. These residual monomers not only compromise the mechanical properties of the adhesive layer but also increase the risk of cytotoxicity, as they can leach into surrounding tissues over time [37]. Furthermore, under-cured adhesives exhibit reduced cohesive strength, lowering the bond strength between the restorative material and the dental substrate. Even minor deviations from optimal curing protocols can significantly weaken the hybrid layer, leading to marginal gap formation, microleakage, and an increased susceptibility to secondary caries [38]. Such outcomes emphasize the necessity for precise control over polymerization parameters, including light intensity, exposure time, and the wavelength of the curing unit, to ensure adequate curing.

The susceptibility of under-cured adhesives to hydrolytic and enzymatic degradation further exacerbates the problem, jeopardizing the longevity of dental restorations. When polymerization is incomplete, the adhesive layer becomes more porous, allowing for water ingress and hydrolytic breakdown of ester bonds within the resin matrix [39]. This process not only deteriorates the adhesive interface but also facilitates the activation of matrix metalloproteinases (MMPs) in dentin, accelerating collagen degradation within the hybrid layer [10]. Recent advances in adhesive technology, such as light-curable systems with photoinitiators that respond to multiple wavelengths or dual-cure systems, have sought to address these issues [40]. However, adherence to strict clinical protocols remains indispensable to mitigate the risks associated with under-curing and to optimize the performance of adhesive systems in restorative dentistry [41].

### 3.2. Biofilm Formation and Bacterial Degradation

The acquired salivary pellicle, a thin organic film that rapidly forms on exposed oral surfaces, plays a critical role in mediating interactions between restorative materials and the oral environment. As the initial layer preceding biofilm formation, the pellicle acts as a selective barrier while also influencing the chemical stability of the adhesive interface. These adverse effects are linked to both acid-induced resin hydrolysis and the enzymatic degradation of collagen fibrils within the hybrid layer. This is a continuous process, which ultimately compromises adhesive bond integrity, increasing the risk of restoration failure and highlighting the need for strategies that mitigate biofilm-associated degradation within the oral environment. Its composition, predominantly rich in proteins such as mucins, proline-rich proteins, and statherin, influences its ability to serve as a selective barrier against external challenges [42]. Studies have shown that the pellicle reduces demineralization by buffering acids and regulating microbial adhesion while also acting as a potential enzymatic reservoir capable of degrading adhesive interfaces [43]. Recent investigations are advancing this understanding by evaluating how the salivary pellicle interacts with material surfaces in microcosm models, particularly in biofilm formation [44,45,46]. For example, the ranking of antimicrobial activity of a ZnO-containing adhesive was significantly affected by the presence of acquired pellicle [45]. These findings emphasize the necessity of considering the acquired pellicle not only as a protective barrier but also as an active factor influencing the long-term integrity of dental biomaterials, highlighting its implications on material design criteria [47].

Once the biofilm is established, acidogenic and acid-tolerant bacteria within it, particularly species such as *Streptococcus mutans*, directly contribute to localized acidity, resulting in a microenvironment that accelerates degradation [48]. The metabolic byproducts of biofilm bacteria not only generate acids that erode the mineralized structures of the tooth, including the hybrid layer if a gap exists between the tooth and the restoration, but also facilitate the penetration of additional degradative agents, further weakening the adhesive interface. The acidic conditions created by biofilms also trigger enzymatic activity, notably by activating matrix metalloproteinases (MMPs), which degrade collagen fibrils in the hybrid layer, thereby undermining the structural stability of the adhesive interface [49]. In vitro studies have demonstrated that adhesive bonds exposed to biofilm conditions exhibit markedly reduced bond strength and heightened microleakage compared to those not exposed to biofilms [30].

### 3.3. Collagen Degradation by Enzymatic Activity

**Figure 3:**
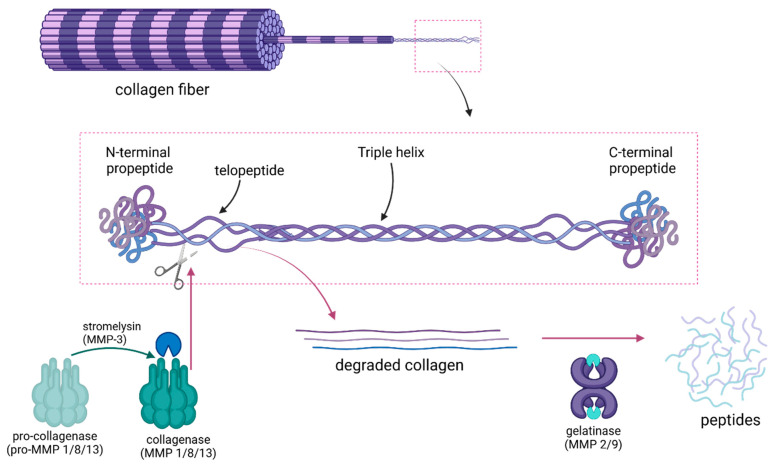
Collagen structure showing the cleavage site for collagenases at the telopeptide region. Pro-collagenases are activated by stromelysin into collagenases, which cleave the triple helix into smaller segments. These segments are then prone to degradation by gelatinases, resulting in the formation of peptides.

Collagen degradation within the hybrid layer is predominantly driven by matrix metalloproteinases (MMPs), a group of zinc-chelating enzymes naturally present in the dentin matrix. In healthy dentin, MMPs typically remain inactive, but they become activated during the bonding process due to the acidic environment introduced by etching and adhesive application. Once activated, MMPs target the exposed collagen fibrils, progressively breaking down the collagen structure over time [50]. Specifically, pro-collagenases such as MMP-1, MMP-8, and MMP-13, once activated by stromelysin (MMP3) into collagenases, initiate degradation by cleaving collagen molecules at specific sites within the triple helix structure, typically located within the telopeptide regions, which are the unstructured regions of collagen at the ends of the helical structure (the N- and C-terminal extensions) (Figure 3). Collagenases cleave at the triple-helix structure rather than simply attacking the termini. The enzyme recognizes and binds to a specific peptide sequence in the collagen structure, and once bound, it hydrolyzes the collagen molecule into smaller fragments and intermediate degradation products [51]. These partially denatured collagen fragments then serve as substrates for gelatinases (MMP-2 and MMP-9), as well as cysteine cathepsins, which further degrade the partially cleaved collagen [36]. Cathepsins, particularly cysteine cathepsins, exhibit optimal activity under acidic conditions, complementing MMPs and amplifying collagen degradation within the hybrid layer [52]. The synergy between MMPs and cathepsins weakens the adhesive interface and makes it increasingly susceptible to further degradation by moisture, bacteria, and other oral agents. This interaction is clinically significant because it ultimately leads to the development of gaps and weak points within the hybrid layer, facilitating infiltration by external agents and initiating a cascade of degradation.

### 3.4. Hydrolytically Derived Degradation of the Adhesive Material

**Figure 4:**
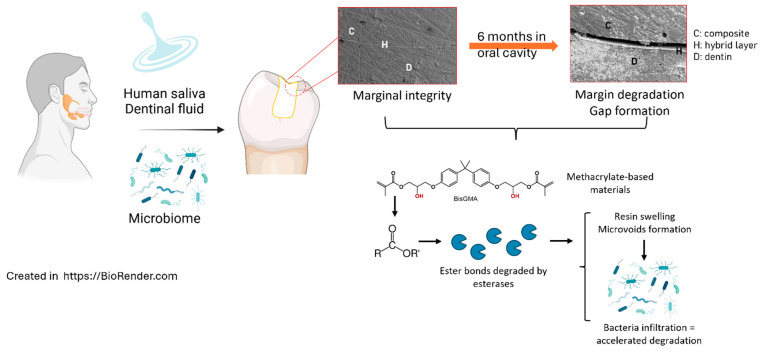
Representation of the hydrolytic and enzymatic degradation sustained by methacrylate-based materials in the oral cavity in the presence of human saliva and oral microbiome.

Hydrolytic degradation poses a significant challenge to the longevity of adhesive bonds in dental restorations. When water infiltrates the resin matrix within the hybrid layer, it initiates the hydrolysis of ester bonds commonly found in methacrylate-based adhesives. This reaction progressively weakens the polymer network, reducing bond strength and increasing the likelihood of mechanical failure over time. Water absorption, sourced from saliva or dentinal fluid, further exacerbates the issue by causing resin swelling. This swelling disrupts the resin–collagen interface, creating microvoids that facilitate additional water ingress and accelerate degradation [40,53]. In addition to weakening the polymer matrix, hydrolytic degradation results in the release of low-molecular-weight byproducts, some of which have been shown to exhibit cytotoxic effects. These degradation byproducts can also promote Streptococcus mutans upregulation, exacerbating biofilm formation on restorative materials [54]. The production of these byproducts and the rate of hydrolysis are pH-dependent, with acidic conditions, such as those created in biofilm-rich environments, serving to amplify the degradation process [55]. An overview of the causes of degradation of the material is shown in Figure 4.

Esterases and pseudocolinesterases present in saliva and dentinal fluid further contribute to hydrolytic degradation by catalyzing the breakdown of ester bonds in methacrylate-based adhesives [56]. These enzymatic reactions significantly accelerate the degradation process, particularly in simplified adhesive systems that incorporate hydrophilic monomers to combine primer and bond steps. The high water affinity of these monomers leads to resin plasticization, dimensional instability, and compromised mechanical properties over time [57]. Collectively, these factors underscore the importance of developing water-resistant adhesive formulations. Current research focuses on reducing hydrophilic monomer content [58], incorporating alternative chemistries [59], and targeting the inhibition of enzymatic activity to enhance hydrolytic stability [60,61]. These strategies aim to mitigate the deleterious effects of water and enzymatic action on adhesive interfaces, extending the functional lifespan of restorations.

## 4. Current Strategies for Enhancing Interfacial Integrity

### 4.1. Antimicrobial Approaches

To improve interfacial integrity and extend the lifespan of dental restorations, antimicrobial strategies have become a central focus in the development of adhesives and composites. There are several examples of compounds that are directly incorporated into the material to exert antibacterial activity, with varied degrees of success. For example, chlorhexidine, a widely used antiseptic, has been explored for incorporation into dental adhesives due to its dual ability to inhibit matrix metalloproteinases (MMPs) and bacterial growth [49]. Applied as a primer or as part of the adhesive formulation, chlorhexidine can help prevent biofilm formation at the adhesive interface, thereby protecting the hybrid layer from both bacterial acids and enzymatic degradation. However, the antimicrobial effects of chlorhexidine may be temporary, as its activity diminishes over time, especially in the moist oral environment [62]. Another example: silver nanoparticles (AgNPs) are known for their broad-spectrum antibacterial properties, and work by releasing silver ions, which penetrate bacterial cell membranes, disrupt metabolic functions, and ultimately cause cell death [63,64]. When incorporated into adhesives, silver nanoparticles create a durable antimicrobial barrier that prevents bacterial growth on the restoration surface [39]. Research indicates that adhesives containing AgNPs exhibit reduced biofilm formation without significantly altering the mechanical properties of the adhesive system. However, challenges such as potential cytotoxicity and discoloration have prompted further refinements to ensure biocompatibility and esthetic outcomes [65].

Quaternary ammonium methacrylates (QAMs) are among the most extensively investigated antimicrobial agents in dental materials. These compounds possess a long alkyl chain with a positive charge, enabling interaction with the negatively charged bacterial cell walls. This interaction disrupts the bacterial membrane, leading to cell lysis and death. Because of the methacrylate functionality, QAMs can be polymerized within the resin matrix, providing sustained antimicrobial properties, not dependent on the leaching of the compound over time. Studies demonstrate that QAM-modified adhesives effectively reduce bacterial colonization on the adhesive surface, thereby minimizing biofilm formation and the subsequent acid and enzyme production that would otherwise compromise the hybrid layer [66].

One caveat of these existing approaches is that they are all based on broad spectrum antibiotics [67]. While broad-spectrum antibiotics incorporated into dental materials, such as quaternary ammonium compounds (QACs) and polymerizable antibiotics like ciprofloxacin, effectively reduce bacterial activity through mechanisms such as cell membrane disruption, they exhibit notable limitations. Broad-spectrum antimicrobial agents indiscriminately target both pathogenic and commensal bacteria, potentially disrupting the oral microbiome’s ecological balance. This disruption may lead to dysbiosis, which is linked to increased susceptibility to secondary oral infections and caries recurrence [68]. Furthermore, broad-spectrum antibiotics often result in the development of bacterial resistance over time, a phenomenon that is particularly concerning in polymicrobial communities, which inherently exhibit enhanced resistance compared to single-species biofilms. Additionally, antimicrobial materials reliant on the release of bioactive agents face challenges regarding sustainability and effectiveness. Apart from the obvious concern with reduced bulk mechanical integrity of the materials [69], other biologically derived concerns arise. For example, biofilm-produced extracellular polymeric substances (EPS) can sequester released antimicrobial compounds, reducing their efficacy at the bacterial cell wall level and allowing biofilm maturation to proceed uninhibited [68]. Moreover, these strategies may create layers of dead bacteria on the material’s surface, which, rather than preventing further colonization, can act as a foundation for subsequent biofilm development. Therefore, while broad-spectrum antibiotics in dental composites offer initial antimicrobial benefits, they underscore the need for targeted approaches that can selectively inhibit pathogenic species while preserving or even promoting commensal bacteria essential for oral health. Emerging strategies, such as surface-modified materials with antifouling properties or biomaterials leveraging localized and contact-dependent mechanisms, hold promise for addressing these limitations effectively [68].

Given the limitations associated with broad-spectrum antimicrobial agents, including the risk of bacterial resistance and disruption of the oral microbiome, an emerging approach is the development of antifouling materials [70]. Unlike traditional antimicrobial strategies, antifouling materials prevent the initial adhesion of microorganisms to restorative surfaces, thereby reducing biofilm formation without directly targeting bacterial viability [70,71]. This strategy involves engineering surfaces with chemical or physical modifications that repel bacterial adhesion. For instance, surfaces functionalized with zwitterionic monomers, such as 2-methacryloyloxyethyl phosphorylcholine (MPC), have demonstrated the ability to inhibit bacterial attachment effectively while preserving the ecological balance of the oral microbiome [72,73]. Zwitterionic coatings can significantly reduce bacterial adhesion and biofilm formation by creating hydrophilic surfaces that resist protein adsorption and microbial colonization. Additionally, these modifications do not compromise the mechanical properties of the adhesive system, making them a promising alternative for enhancing the durability of the hybrid layer [74,75]. By mitigating bacterial colonization without the selective pressure associated with antimicrobial agents, antifouling materials represent a novel avenue for improving the longevity and performance of dental restorations.

### 4.2. Collagen Stabilization Techniques

Several approaches have been developed to reinforce and/or prevent degradation of collagen networks, and these fall into two main categories: 1. collagen crosslinking, which increases stiffness of the network and makes it less prone to degradation; 2. Inhibition of collagen degrading enzymes, or a combination of the two [40]. These will be explored in more detail in the following sections.

### 4.3. Collagen Crosslinking Agents

There are several different classes of compounds that have been proposed to reinforce collagen either via secondary intermolecular interactions or covalent crosslinking. The former is usually accomplished with compounds capable, for example, of strong hydrogen bonding interactions, such as phenolic compounds [76]. The latter relies on tissue fixators, such as glutaraldehyde or chlorhexidine [77]. Glutaraldehyde, a potent crosslinking agent, has been widely studied for its ability to enhance the mechanical stability of resin–dentin bonds by stabilizing the collagen matrix. Its bifunctional aldehyde groups react with amino groups in collagen, forming covalent crosslinks that increase the structural integrity of the dentin matrix and reduce its susceptibility to enzymatic degradation. This stabilization inhibits the activity of matrix metalloproteinases (MMPs), preserving the hybrid layer over time [78]. Studies have demonstrated that glutaraldehyde-containing agents effectively reduce proteolytic activity and enhance the longevity of adhesive interfaces. Pre-treatment of etched dentin with glutaraldehyde solutions has been shown to improve mechanical stability and reduce enzymatic degradation of the resin–dentin bond after prolonged storage periods [79]. These findings highlight the potential of glutaraldehyde to significantly delay the degradation of the hybrid layer. Chlorhexidine offers dual benefits by both inhibiting MMP activity, as will be discussed in more detail later, and facilitating mild, indirect crosslinking within the collagen network [80], though not as effectively as glutaraldehyde or other known crosslinkers such as Carbodiimide (EDC). EDC is an effective crosslinking agent, which stabilizes collagen by forming peptide bonds within the fibrils, reducing the sites available for enzymatic cleavage. This crosslinking effect has been shown to maintain the hybrid layer’s stability, preserving bond strength and reducing microleakage over time. EDC-treated dentin demonstrates enhanced resistance to both hydrolytic and enzymatic degradation, helping to prevent the weakening of the adhesive bond [81].

In the case of compounds that exert their effect via secondary intermolecular interactions, phenolic compounds such as proanthocyanidins, which are natural compounds derived from sources such as grape seed extract, have shown strong potential as collagen crosslinkers [82]. Proanthocyanidins (PACs) stabilize collagen by forming covalent bonds within collagen fibrils, reinforcing the triple-helix structure and enhancing mechanical stability. This stabilization improves collagen’s resistance to enzymatic degradation, particularly by matrix metalloproteinases (MMPs), while also enhancing bond strength and the durability of the adhesive interface. Studies have reported that adhesives supplemented with PACs significantly reduce collagen degradation and exhibit improved long-term bond performance compared to untreated adhesives [76]. More recent in depth studies of the interactions of proantocyanidins with collagen type I have demonstrated that the specific interflavan linkages present in the molecules significantly impact their bioactive conformations, with molecules with B-type and complex AB-type linkages providing the best candidates for dentin biomodification [83]. Further, catechin (C) and epicatechin (EC) terminal units have also been demonstrated to have distinct modulatory effects of proantocyanidins and collagen on the stiffness of the dentin matrix at different length scales [84]. Genipin, another naturally derived crosslinker, has gained attention for its low cytotoxicity and strong collagen-stabilizing properties. While genipin showed potential in enhancing the enzymatic resistance of the collagen matrix, studies have demonstrated that it does not significantly improve the adhesive bond strength to dentin. In comparison to other cross-linkers such as glutaraldehyde and grape seed extract, genipin’s effects on bond strength were limited. Additionally, practical challenges, including prolonged application times and potential discoloration of treated tissues, have further restricted its clinical applicability. As a result, genipin has not established itself as a viable solution for improving dentin bond strength in adhesive dentistry [85].

Recent studies have highlighted the potential of multi-acrylamide compounds to enhance the durability of dentin-bonded interfaces by reinforcing the collagen matrix. These monomers, such as tri- and di-tertiary acrylamides, have been shown to interact covalently with collagen fibrils, providing mild crosslinking that increases the mechanical stability of the hybrid layer [14]. This stabilization reduces enzymatic hydrolysis under physiological conditions, as demonstrated by the significant reduction in hydroxyproline release in treated samples [86]. Additionally, adhesives incorporating these compounds exhibit improved shear storage modulus and resistance to hydrolytic degradation, ensuring hybrid layer stability even after extended exposure to moist environments [14]. Complementary findings using spectroscopic analysis confirm that multi-acrylamides induce partial collagen denaturation and promote crosslink formation, reinforcing the collagen network against both enzymatic and hydrolytic challenges [86]. These monomers also display reduced polymerization shrinkage compared to methacrylate-based adhesives, minimizing the formation of interfacial gaps and contributing to improved long-term performance of adhesive restorations [14]. Together, these properties position multi-acrylamides as promising agents to address critical limitations of conventional adhesive systems.

### 4.4. Enzyme Inhibitors

Matrix metalloproteinase (MMP) inhibitors have been widely studied for their role in preserving the collagen matrix within the hybrid layer. Chlorhexidine, a prominent example in dentistry, inhibits MMP activity by chelating zinc ions at their active site, thereby reducing enzymatic degradation of collagen. Applied as a dentin pre-treatment before adhesive application, chlorhexidine has shown efficacy in maintaining bond strength over time [87,88]. Studies confirm that chlorhexidine effectively preserves the resin–dentin interface, even after artificial aging, by inhibiting key MMPs such as MMP-2, MMP-8, and MMP-9. However, its inhibitory effects diminish under prolonged water storage or thermal cycling, conditions that simulate the oral environment [89]. In vivo studies further demonstrate its potential to arrest subclinical degradation of hybrid layers in dentin, though questions remain about its long-term efficacy [77]. Tetracyclines, particularly doxycycline, are another well-documented group of MMP inhibitors. Doxycycline acts by chelating zinc ions necessary for MMP activation, thereby reducing enzymatic activity. Non-antibiotic formulations of doxycycline have demonstrated effectiveness in preserving the hybrid layer without contributing to antimicrobial resistance [90]. Clinical studies show that dentin treated with doxycycline exhibits reduced collagen degradation and enhanced long-term bond strength.

In addition to synthetic inhibitors like chlorhexidine and tetracyclines, natural compounds have emerged as promising alternatives for inhibiting MMP activity while offering lower cytotoxicity and additional therapeutic benefits [13]. Polyphenols, including epigallocatechin gallate (EGCG) from green tea and proanthocyanidins from grape seed extract, are among the most extensively studied natural inhibitors. EGCG inhibits MMP-2 and MMP-9 activity while providing antioxidant protection, thereby mitigating oxidative stress-induced collagen breakdown [91]. These compounds also exhibit antioxidant properties, adding another layer of protection against oxidative stress. Curcumin, the active compound in turmeric, has been shown to inhibit MMP activity through the suppression of NF-κB signaling pathways, offering both anti-inflammatory and antioxidant benefits [92]. Similarly, resveratrol, a polyphenol found in red wine and grapes, has demonstrated the ability to inhibit MMP-9 activity and reduce oxidative stress, further contributing to hybrid layer stability [93]. Galardin, a synthetic broad-spectrum MMP inhibitor, has shown potent effects in reducing collagen degradation within the hybrid layer. Studies demonstrate that Galardin significantly decreases MMP-mediated degradation, providing long-lasting preservation of collagen fibrils. However, its clinical application is limited by challenges in formulation stability and potential biocompatibility issues, necessitating further development [10]. While these natural compounds show promise, challenges related to their bioavailability and integration into dental adhesives remain areas of active research.

Finally, some studies suggest that quaternary ammonium compounds (QAMs) not only exhibit antimicrobial properties but also inhibit MMP activity, although at higher concentrations than chlorhexidine [94]. By integrating these dual-function compounds into adhesive systems, it may be possible to achieve synergistic effects that further protect the hybrid layer.

### 4.5. Hydrolytically Resistant Materials

The hybrid layer in adhesive systems is continuously exposed to moisture in the oral environment, posing a significant risk to the longevity of these restorations. The phenomenon of “water treeing” leads to degradation over storage periods as short as six months [95]. While the approaches described earlier in this review focused on direct collagen preservation, recent advancements have shifted toward the development of materials that resist hydrolysis, either by water at low pH or through the action of esterases [57].

One approach to creating water-resistant materials is to reduce or eliminate hydrophilic monomers in adhesive formulations. Traditional adhesives often contain hydrophilic monomers like HEMA (2-hydroxyethyl methacrylate), which assist with resin infiltration into the dentin’s collagen network. These monomers readily absorb water, leading to resin swelling, hydrolytic breakdown, and microleakage [57,95]. Newer adhesive systems aim to replace HEMA with more hydrophobic alternatives or reduce the overall hydrophilic component, thus, decreasing water uptake and enhancing the adhesive resistance to water degradation [89]. Examples include methacryloxyethyl trimethyl silane (MES), and tetrahydroxy dimethacrylate (THDMA), a monomer derived from tartaric acid [20]. Substituting HEMA with THDMA in adhesive formulations results in higher conversion rates and improved mechanical properties, such as increased flexural modulus and strength, and lower water uptake and solubility [96].

Additionally, ester-free alternatives have been investigated. For example, acrylamide-based adhesives have shown promise as a robust alternative to traditional methacrylate-based adhesives. By incorporating amide bonds, which are less susceptible to water-induced breakdown, these adhesives provide improved stability in moist environments. These adhesives have demonstrated superior long-term bond strength and reduced degradation compared to conventional methacrylate-based adhesives, particularly in high-moisture conditions [59]. At least one commercial product incorporates acrylamide monomers to reduce water uptake and improve resistance to hydrolytic degradation. Clinical evaluations have demonstrated its durability, showing long-term stability in adhesive restorations, particularly in challenging oral environments [26,59,96]. However, these materials typically require co-polymerization with methacrylates due to their inherent water uptake and loss of mechanical properties after water storage [26]. Other examples include vinyl ether-based materials, which have shown great stability when subjected to enzymatic challenge, with multifunctional monomers leading to materials with mechanical properties comparable to methacrylate controls [97]. One drawback of vinyl ethers, in general, is that their homopolymerization is very slow at room temperature, requiring either specialized initiators, higher temperatures, or co-polymerization with more reactive species such as methacrylates [97]. Others have proposed using azide-alkyne click polymerizations, which produce polymers with high Tg and toughness and lower polymerization stress. These materials require a copper catalyst but can be photoactivated, and they are completely ester-free [98]. Potential pitfalls lower mechanical properties and issues associated with the presence of copper, even at minute concentrations, such as toxicity and discoloration. Another example is thiol vinyl sulfones, which polymerize via Michael and can reach 80% conversion with Tg as high as 100 °C [99,100]. These are also ester-free, are extremely resistant to solvent degradation, and can be photoactivated using a special photobase generator [99,100].

## 5. Emerging Trends and Future Directions

Advances in degradation-resistant polymers, self-healing materials, and bioactive resins hold promise for significantly improving the performance and durability of dental restorations. These innovations target the root causes of adhesive degradation, offering solutions that address not only hydrolytic and enzymatic challenges but also introduce new functionalities to support long-term restoration success.

### 5.1. Self-Healing Materials

Self-healing materials represent an innovative approach in restorative dentistry, designed to autonomously repair microdamage within the adhesive interface, thereby enhancing the longevity of restorations. These materials work through mechanisms such as the release of healing agents from microcapsules or the activation of dynamic covalent bonds in response to stress or fracture. For example, microcapsule-based systems contain polymerizable healing agents, such as triethylene glycol dimethacrylate (TEGDMA), which are released when microcracks form. Once released, these agents polymerize and restore the structural integrity of the adhesive bond. Research has demonstrated that composites with embedded microcapsules exhibit significant recovery in fracture toughness after damage, indicating their potential for self-repair [101,102,103,104]. Another approach involves dynamic covalent bonds, such as Diels–Alder networks, which can reversibly break and reform under specific stimuli like heat or light. This mechanism allows materials to self-heal multiple times, maintaining the integrity of the adhesive interface even under repeated mechanical stresses [105,106,107]. Integrating such materials into clinical practice holds significant promise for extending restoration lifespans by preventing minor damage from evolving into major failures. Continued research is essential to optimize these systems for real-world dental applications, ensuring they function effectively under the complex conditions of the oral environment.

Emerging materials have demonstrated potential for addressing the challenges of hybrid layer degradation. However, understanding the interaction between these materials and dentin substrates is critical for optimizing their clinical performance [108,109]. One comprehensive systematic review and meta-analysis highlighted that moisture control significantly influences the hybrid layer’s integrity and the adhesive bond strength, with the main message being that proper moisture management enhances resin infiltration into demineralized dentin, reducing the risk of nanoleakage and enzymatic degradation [108]. Another review systematically evaluating self-etch adhesives revealed that deproteinization of the hybrid layer improves the interaction between adhesive resins and dentin by removing denatured collagen and exposing the underlying substrate. This process facilitates better chemical and micromechanical adhesion, ultimately enhancing bond durability. These findings suggest that modifying the smear layer could complement the design of self-healing adhesives by improving their interaction with dentin and resistance to degradation over time [109].

### 5.2. Bioactive Adhesives

Bioactive dental materials are those that interact with biological systems to promote some sort of response, such as remineralization of tooth structures, tissue regeneration, or prevention of decay. These materials can release ions such as calcium, phosphate, and fluoride, or form superficial layers that enhance biological processes, such as cell attachment [110]. Examples include glass ionomer cements and bioactive composites, which help promote the healing of surrounding tissues or mineralize enamel. Another example, β-TCP acts as a reservoir for calcium and phosphate ions, facilitating the remineralization of demineralized dentin and aiding in the preservation of the hybrid layer [111,112]. Similarly, calcium silicate materials, such as Biodentine, also release calcium ions, which promote hydroxyapatite formation and dentin remineralization. Although primarily used as restorative materials, the principles underlying calcium silicate bioactivity inspire the development of adhesive systems that create a remineralizing microenvironment [113]. A novel adhesive formulation incorporating dimethylaminohexadecyl methacrylate (DMAHDM) and calcium phosphate nanoparticles (CaP NPs) demonstrated dual bioactivity by simultaneously inhibiting matrix metalloproteinases (MMPs) and releasing remineralizing ions, effectively stabilizing the hybrid layer. Over three months of aging in artificial saliva, the adhesive exhibited resistance to nanoleakage and maintained bond strength, highlighting its capacity to counteract degradation and preserve the adhesive-dentin interface under simulated clinical conditions [114]. Other bioactive restorative materials with integrated antibacterial agents and their effects on dentin bond strength have been evaluated [115]. These materials were designed to release therapeutic ions such as calcium, phosphate, or fluoride, fostering remineralization while simultaneously preventing biofilm formation. Importantly, the study demonstrated that incorporating antibacterial agents did not compromise dentin bond strength, a critical consideration for clinical applications.

Other strategies have also been proposed, such as the incorporation of farnesol and bioactive glass, which aim to enhance the longevity and bioactivity of dental restorations. Farnesol, a naturally occurring sesquiterpene alcohol, has demonstrated antimicrobial properties when incorporated into dental adhesives, and it has been shown to reduce biofilm formation and bacterial growth at the interface between the tooth and the restoration, thus, potentially helping to prevent secondary caries and enhance the overall durability of the restoration [116]. Furthermore, farnesol may also contribute to mineralization of dentin, mimicking the natural remineralization processes of teeth. The antimicrobial and bioactive properties of farnesol, when combined with adhesive systems, can significantly improve both the functional and therapeutic performance of dental restorations. In addition, to improve substantivity and achieve longer release, farnesol has been loaded into bioactive glass (BAG), which has the added benefit of compounding farnesol’s antimicrobial properties with BAG’s ability to release calcium, phosphate, and fluoride ions, which promote remineralization of the tooth structure. This approach enhances the adhesive interface by forming a hydroxyapatite-like layer that improves the bond strength and reduces the risk of restoration failure. This material has been particularly effective in providing long-term protection against demineralization and enhancing the reparative capacity of dentin, using a multifunctional approach to dental adhesion that can address both structural and biological needs [117].

Finally, it must be pointed out that the definition of bioactivity is not universally agreed upon, and it is a complex, multidimensional concept. Some scholars caution against over-interpretation, noting that bioactivity should be seen as the ability of a material to interact with biological systems in ways that promote health, without overestimating its capacity to prevent or cure all forms of damage [118]. Materials must demonstrate clinically relevant effects, such as supporting remineralization or stimulating biological responses, to be considered bioactive. While bioactive materials have the potential to improve long-term dental health, the field remains nuanced and requires careful consideration of material properties and biological interactions [119].

### 5.3. Peptide-Based Systems

A significant emerging trend in restorative dentistry is the development of bioactive peptide-based systems, some of which work as dual-function coatings that combine hydrophobic modification with antimicrobial properties, establishing a two-tier protective system for the dentin-restoration interface. These coatings reduce water infiltration and shield the interface from hydrolytic and enzymatic degradation, key factors contributing to adhesive failure. Concurrently, their antimicrobial properties inhibit biofilm formation, effectively lowering the risk of secondary caries [120]. Furthermore, peptide-based biomaterials such as P11-4 represent a novel approach to biomimetic remineralization. Designed to promote the recovery of hydroxyapatite crystals within carious lesions, P11-4 has shown promise in initial enamel caries. Its applicability to dentin is gaining attention, as studies demonstrate its interaction with collagen type I fibers. P11-4 binds to these fibers, increasing their width and resistance to proteolytic degradation by collagenases, thereby enhancing the stability of the hybrid layer. Additionally, immediate application of P11-4 to artificial caries-affected dentin has been shown to significantly improve the microtensile bond strength, achieving values similar to sound dentin. However, its effects diminish after six months of water storage, indicating potential limitations in long-term stability [121].

## 6. Limitations of the Studied Approaches

Methodological limitations can profoundly impact the validity and applicability of study outcomes, particularly in adhesive dentistry research. Techniques such as static immersion models or in situ zymography, while informative, often fail to replicate the complex, dynamic oral environment, potentially leading to overestimation or underestimation of material performance. Similarly, artifacts introduced by sample preparation for microscopy or the reliance on simplified substrates may distort the interpretation of structural integrity and enzymatic activity at the resin–dentin interface. These methodological constraints can result in findings that, while insightful, may lack translational relevance or predictive accuracy for clinical settings, underscoring the need for more robust, physiologically relevant models.

Pioneering studies using qualitative analysis through scanning electron microscopy (SEM) laid the foundation for the hybrid layer concept [122]. Later studies using transmission electron microscopy (TEM) significantly advanced our understanding of the resin–dentin interface [123]. Some limitations of these methods include the need for ultrathin sections and dehydration protocols, which introduce potential artifacts, complicating the interpretation of the adhesive interface’s morphology. Moreover, the inherently small sample size and narrow field of view in TEM limited the generalizability of their findings to broader clinical applications. In situ zymography has been employed to elucidate matrix metalloproteinase (MMP) activity within the hybrid layer, providing valuable insights into enzymatic degradation [124]. Others have used advanced analytical techniques such as mass spectrometry and biodegradation assays to evaluate the degradation of adhesive interfaces [54]. While these approaches provide critical insights, the complex oral environment milieu, with fluctuations in temperature and pH, as well as salivary flow and clearance, which are in turn affected by mechanical stress and biofilm-derived enzymatic activity, makes it difficult to extrapolate results to clinical situations. Specifically for the dentin, current methods only approximate native collagen, and they may not fully capture the kinetics and specificity of MMP activity in vivo. Methods that are able to quantify the degree of resin infiltration or detect void distribution within the hybrid layer comprehensively, in a dynamic rather than static manner, were later developed in an attempt to replicate the complex biomechanical and biochemical challenges present in the oral environment, such as cyclic loading and enzymatic degradation over time. Others have also introduced bioreactor-type devices, which provide a step in the right direction, but they are still limited, often replicating only two or three physiological factors (bacteria and mechanical load, for example) [125]. More recently, in vivo caries models have also been reported, where technically all factors would be replicated. However, rat models, for example, have a very different native oral microflora, which means they need to be inoculated with bacteria relevant to the human oral environment, which in turn might not develop biofilms the same way in a different mammalian species [126,127]. The main take-home message here is that while clinical studies, in particular practice-based clinical studies, provide the best evidence, and the aforementioned methods provide a reasonable means for screening of materials.

## 7. Conclusions

Preserving interfacial integrity in dental restorations continues to be a significant challenge due to the hybrid layer’s susceptibility to enzymatic, hydrolytic, and microbial degradation. While advances in adhesive technologies, such as enzymatic inhibitors, crosslinking agents, and hydrolysis-resistant materials, have improved bond stability, the hybrid layer retains inherent vulnerabilities that affect long-term clinical success. Emerging bioactive materials, including self-healing adhesives and bioactive resins, offer promising approaches by introducing functionalities that actively resist degradation and potentially contribute to interfacial repair. The development of adhesive systems that effectively address the multifactorial challenges of the oral environment will require ongoing innovation in both materials science and clinical application.

Looking ahead, the integration of advanced technologies into multifunctional adhesive systems represents an exciting frontier in dental materials science. Researchers are developing hybrid adhesives that combine inhibitors, antimicrobial properties, and moisture resistance, aiming to create restoratives that not only prevent degradation but also actively repair and protect the tooth-restoration interface. Furthermore, the application of nanotechnology is enhancing the dispersion and effectiveness of bioactive and antimicrobial agents within the resin matrix, which may significantly increase the longevity and reliability of these new materials. By effectively addressing core challenges such as moisture, enzymatic activity, and bacterial colonization, these innovative materials have the potential to usher in a new generation of adhesive systems that provide durable, self-sustaining protection for dental restorations, ultimately improving long-term patient outcomes.

## Data Availability

No new data were created or analyzed in this study. Data sharing is not applicable to this article.

## References

[1] Jokstad A. (2016). Secondary caries and microleakage. Dent. Mater..

[2] Nakabayashi N. (1992). The hybrid layer: A resin-dentin composite. Proc. Finn. Dent. Soc..

[3] Van Meerbeek B., Inokoshi S., Braem M., Lambrechts P., Vanherle G. (1992). Morphological aspects of the resin-dentin interdiffusion zone with different dentin adhesive systems. J. Dent. Res..

[4] Carvalho R.M., Manso A.P., Geraldeli S., Tay F.R., Pashley D.H. (2012). Durability of bonds and clinical success of adhesive restorations. Dent. Mater..

[5] Tjäderhane L., Nascimento F.D., Breschi L., Mazzoni A., Tersariol I.L., Geraldeli S., Tezvergil-Mutluay A., Carrilho M., Carvalho R.M., Tay F.R. (2013). Strategies to prevent hydrolytic degradation of the hybrid layer-A review. Dent. Mater..

[6] Ferracane J.L. (2024). A Historical Perspective on Dental Composite Restorative Materials. J. Funct. Biomater..

[7] Fugolin A.P.P., Costa A.R., Correr-Sobrinho L., Crystal Chaw R., Lewis S., Ferracane J.L., Pfeifer C.S. (2021). Toughening and polymerization stress control in composites using thiourethane-treated fillers. Sci. Rep..

[8] de Lucena F.S., Lewis S.H., Fugolin A.P.P., Furuse A.Y., Ferracane J.L., Pfeifer C.S. (2022). Triacrylamide-Based Adhesives Stabilize Bonds in Physiologic Conditions. J. Dent. Res..

[9] Askar H., Krois J., Göstemeyer G., Bottenberg P., Zero D., Banerjee A., Schwendicke F. (2020). Secondary caries: What is it, and how it can be controlled, detected, and managed?. Clin. Oral Investig..

[10] Mazzoni A., Tjäderhane L., Checchi V., Di Lenarda R., Salo T., Tay F.R., Pashley D.H., Breschi L. (2015). Role of dentin MMPs in caries progression and bond stability. J. Dent. Res..

[11] Mazzoni A., Scaffa P., Carrilho M., Tjäderhane L., Di Lenarda R., Polimeni A., Tezvergil-Mutluay A., Tay F.R., Pashley D.H., Breschi L. (2013). Effects of etch-and-rinse and self-etch adhesives on dentin MMP-2 and MMP-9. J. Dent. Res..

[12] De Munck J., Van den Steen P.E., Mine A., Van Landuyt K.L., Poitevin A., Opdenakker G., Van Meerbeek B. (2009). Inhibition of enzymatic degradation of adhesive-dentin interfaces. J. Dent. Res..

[13] Nair D.P., Asby S., de Lucena F.S., Pfeifer C.S. (2024). An introduction to antibacterial materials in composite restorations. JADA Found. Sci..

[14] Scaffa P.M.C., Logan M.G., Icimoto M.Y., Fugolin A.P.P., Tsuzuki F.M., Lewis S.H., Pfeifer C.S. (2024). Mechanistic study of the stabilization of dentin-bonded restorative interfaces via collagen reinforcement by multi-acrylamides. Dent. Mater..

[15] Scaffa P.M.C., Kendall A., Icimoto M.Y., Fugolin A.P.P., Logan M.G., DeVito-Moraes A.G., Lewis S.H., Zhang H., Wu H., Pfeifer C.S. (2023). The potential use of glycosyl-transferase inhibitors for targeted reduction of S. mutans biofilms in dental materials. Sci. Rep..

[16] Lee Y.H., Zimmerman J.N., Custodio W., Xiao Y., Basiri T., Hatibovic-Kofman S., Siqueira W.L. (2013). Proteomic Evaluation of Acquired Enamel Pellicle during In Vivo Formation. PLoS ONE.

[17] Pinna R., Usai P., Filigheddu E., Garcia-Godoy F., Milia E. (2017). The role of adhesive materials and oral biofilm in the failure of adhesive resin restorations. Am. J. Dent..

[18] Marshall G.W., Marshall S.J., Kinney J.H., Balooch M. (1997). The dentin substrate: Structure and properties related to bonding. J. Dent..

[19] Saikaew P., Sattabanasuk V., Harnirattisai C., Chowdhury A.F.M.A., Carvalho R., Sano H. (2022). Role of the smear layer in adhesive dentistry and the clinical applications to improve bonding performance. Jpn. Dent. Sci. Rev..

[20] Breschi L., Maravic T., Cunha S.R., Comba A., Cadenaro M., Tjäderhane L., Pashley D.H., Tay F.R., Mazzoni A. (2018). Dentin bonding systems: From dentin collagen structure to bond preservation and clinical applications. Dent. Mater..

[21] Lu S., Zhao S.J., Gao Y., Sun Y., Li X., Chen J.H. (2014). Proteoglycans affect monomer infiltration in the etch-and-rinse bonding technique. Dent. Mater..

[22] Carrilho E., Cardoso M., Marques Ferreira M., Marto C.M., Paula A., Coelho A.S. (2019). 10-MDP Based Dental Adhesives: Adhesive Interface Characterization and Adhesive Stability-A Systematic Review. Materials.

[23] Betancourt D.E., Baldion P.A., Castellanos J.E. (2019). Resin-Dentin Bonding Interface: Mechanisms of Degradation and Strategies for Stabilization of the Hybrid Layer. Int. J. Biomater..

[24] Frassetto A., Breschi L., Turco G., Marchesi G., Di Lenarda R., Tay F.R., Pashley D.H., Cadenaro M. (2016). Mechanisms of degradation of the hybrid layer in adhesive dentistry and therapeutic agents to improve bond durability—A literature review. Dent. Mater..

[25] Mokeem L.S., Garcia I.M., Melo M.A. (2023). Degradation and Failure Phenomena at the Dentin Bonding Interface. Biomedicines.

[26] Fugolin A.P., Dobson A., Mbiya W., Navarro O., Ferracane J.L., Pfeifer C.S. (2019). Use of (meth)acrylamides as alternative monomers in dental adhesive systems. Dent. Mater..

[27] Fugolin A.P., Lewis S., Logan M.G., Ferracane J.L., Pfeifer C.S. (2020). Methacrylamide-methacrylate hybrid monomers for dental applications. Dent. Mater..

[28] Jafer M.A., Qadiri A.A., Mtwam N.A., Hakami A.H., Mowkly A.A., Bhandi S., Patil S. (2022). Influence of Human and Bacterial Enzymes on Resin Restorations: A Review. J. Contemp. Dent. Pract..

[29] Mahalaxmi S., Madhubala M.M., Jayaraman M., Sathyakumar S. (2016). Evaluation of matrix metalloproteinase and cysteine cathepsin activity in dentin hybrid layer by gelatin zymography. Indian J. Dent. Res..

[30] Tsuzuki F.M., Logan M.G., Lewis S.H., Correr-Sobrinho L., Pfeifer C.S. (2024). Stability of the Dentin-Bonded Interface Using Self-Etching Adhesive Containing Diacrylamide after Bacterial Challenge. ACS Appl. Mater. Interfaces.

[31] Sahadi B.O., Sebold M., André C.B., Nima G., Dos Santos A., Chiari M.D.E.S., Nascimento F.D., Tersariol I.L.D.S., Giannini M. (2024). Effect of experimental dentin etchants on dentin bond strength, metalloproteinase inhibition, and antibiofilm activity. Dent. Mater..

[32] Moon P.C., Weaver J., Brooks C.N. (2010). Review of matrix metalloproteinases’ effect on the hybrid dentin bond layer stability and chlorhexidine clinical use to prevent bond failure. Open Dent. J..

[33] Hashimoto M., Ohno H., Kaga M., Endo K., Sano H., Oguchi H. (2000). In vivo Degradation of Resin-Dentin Bonds in Humans Over 1 to 3 Years. J. Dent. Res..

[34] Spencer P., Ye Q., Misra A., Goncalves S.E.P., Laurence J.S. (2014). Proteins, Pathogens, and Failure at the Composite-Tooth Interface. J. Dent. Res..

[35] Feitosa V.P., Leme A.A., Sauro S., Correr-Sobrinho L., Watson T.F., Sinhoreti M.A., Correr A.B. (2012). Hydrolytic degradation of the resin-dentine interface induced by the simulated pulpal pressure, direct and indirect water ageing. J. Dent..

[36] Kiuru O., Sinervo J., Vähänikkilä H., Anttonen V., Tjäderhane L. (2021). MMP Inhibitors and Dentin Bonding: Systematic Review and Meta-Analysis. Int. J. Dent..

[37] Kurt A., Altintas S.H., Kiziltas M.V., Tekkeli S.E., Guler E.M., Kocyigit A., Usumez A. (2018). Evaluation of residual monomer release and toxicity of self-adhesive resin cements. Dent. Mater. J..

[38] Al-Zain A.O., Albuqayli A., Albogami A., Alkudsi A., Alwabiri M., Koshak A.T., Alsefri H., Munchow E.A. (2024). Effect of double adhesive layer application on micro-tensile dentin bond strength of a universal adhesive. Front. Dent. Med..

[39] Perdigão J., Reis A., Loguercio A.D. (2013). Dentin adhesion and MMPs: A comprehensive review. J. Esthet. Restor. Dent..

[40] Cadenaro M., Josic U., Maravić T., Mazzitelli C., Marchesi G., Mancuso E., Breschi L., Mazzoni A. (2023). Progress in Dental Adhesive Materials. J. Dent. Res..

[41] Cadenaro M., Antoniolli F., Sauro S., Tay F.R., Di Lenarda R., Prati C., Biasotto M., Contardo L., Breschi L. (2005). Degree of conversion and permeability of dental adhesives. Eur. J. Oral Sci..

[42] Siqueira W.L., Zhang W., Helmerhorst E.J., Gygi S.P., Oppenheim F.G. (2007). Identification of protein components in in vivo human acquired enamel pellicle using LC-ESI-MS/MS. J. Proteome Res..

[43] Siqueira W.L., Custodio W., McDonald E.E. (2012). New insights into the composition and functions of the acquired enamel pellicle. J. Dent. Res..

[44] Li F., Weir M.D., Fouad A.F., Xu H.H.K. (2014). Effect of salivary pellicle on antibacterial activity of novel antibacterial dental adhesives using a dental plaque microcosm biofilm model. Dent. Mater..

[45] Garcia I.M., Balhaddad A.A., Ibrahim M.S., Weir M.D., Xu H.H.K., Collares F.M., Melo M.A.S. (2021). Antibacterial response of oral microcosm biofilm to nano-zinc oxide in adhesive resin. Dent. Mater..

[46] Melo M.A.S., Garcia I.M., Mokeem L., Weir M.D., Xu H.H.K., Montoya C., Orrego S. (2023). Developing Bioactive Dental Resins for Restorative Dentistry. J. Dent. Res..

[47] Fischer N.G., Aparicio C. (2021). The salivary pellicle on dental biomaterials. Colloids Surf. B Biointerfaces.

[48] Hao Y., Huang X., Zhou X., Li M., Ren B., Peng X., Cheng L. (2018). Influence of Dental Prosthesis and Restorative Materials Interface on Oral Biofilms. Int. J. Mol. Sci..

[49] Breschi L., Maravic T., Comba A., Cunha S.R., Loguercio A.D., Reis A., Hass V., Cadenaro M., Mancuso E., Mayer-Santos E. (2020). Chlorhexidine preserves the hybrid layer in vitro after 10-years aging. Dent. Mater..

[50] Cui J., Shu H., Gu X., Wu S., Liu X., Cao P. (2024). Enhancing antibacterial performance and stability of implant materials through surface modification with polydopamine/silver nanoparticles. Colloids Surf. B Biointerfaces.

[51] Bohn G., Liden B., Schultz G., Yang Q., Gibson D.J. (2016). Ovine-Based Collagen Matrix Dressing: Next-Generation Collagen Dressing for Wound Care. Adv. Wound Care.

[52] Tersariol I.L., Geraldeli S., Minciotti C.L., Nascimento F.D., Pääkkönen V., Martins M.T., Carrilho M.R., Pashley D.H., Tay F.R., Salo T. (2010). Cysteine Cathepsins in Human Dentin-Pulp Complex. J. Endod..

[53] Costa M.P., Giacomini M.C., Zabeu G.S., Mosquim V., Dallavilla G.G., Santos P.S.D.S., Wang L. (2024). Impact of functional monomers, bioactive particles, and HEMA, on the adhesive performance of self-etch adhesive systems applied to simulated altered dentin. J. Dent..

[54] Bourbia M., Finer Y. (2018). Biochemical Stability and Interactions of Dental Resin Composites and Adhesives with Host and Bacteria in the Oral Cavity: A Review. J. Can. Dent. Assoc..

[55] Mirmohammadi H., Kleverlaan C.J., Aboushelib M.N., Feilzer A.J. (2011). Influence of salivary enzymes and alkaline pH environment on fatigue behavior of resin composites. Am. J. Dent..

[56] Shokati B., Tam L.E., Santerre J.P., Finer Y. (2010). Effect of salivary esterase on the integrity and fracture toughness of the dentin-resin interface. J. Biomed. Mater. Res. Part B Appl. Biomater..

[57] Finer Y., Jaffer F., Santerre J.P. (2004). Mutual influence of cholesterol esterase and pseudocholinesterase on the biodegradation of dental composites. Biomaterials.

[58] Korkmaz B., Demirel E., Ye Q., Misra A., Tamerler C., Spencer P. (2024). Synergistic enhancement of hydrophobic dental adhesives: Autonomous strengthening, polymerization kinetics, and hydrolytic resistance. Front. Dent. Med..

[59] Fugolin A.P.P., Navarro O., Logan M.G., Huynh V., França C.M., Ferracane J.L., Pfeifer C.S. (2020). Synthesis of di- and triacrylamides with tertiary amine cores and their evaluation as monomers in dental adhesive interfaces. Acta Biomater..

[60] Li K., Ngo F.M., Yau A.Y.L., Tam W.W.L., Tse E.C.M., Tsoi J.K.H., Yiu C.K.Y. (2022). Mussel-inspired monomer—A new selective protease inhibitor against dentine collagen degradation. Dent. Mater..

[61] Maske T.T., Kuper N.K., Cenci M.S., Huysmans M.C.D.N.J.M. (2019). Chlorhexidine, a matrix metalloproteinase inhibitor and the development of secondary caries wall lesions in a microcosm biofilm model. Caries Res..

[62] Prasad T., Pawar R., Ganiger C., Ronad Y., Phaphe S., Mane P., Patil S. (2024). The Impact of Orthodontic Adhesive Containing Resveratrol, Silver, and Zinc Oxide Nanoparticles on Shear Bond Strength: An In Vitro Study. Cureus.

[63] Wang J., Jiang W., Liang J., Ran S. (2022). Influence of silver nanoparticles on the resin-dentin bond strength and antibacterial activity of a self-etch adhesive system. J. Prosthet. Dent..

[64] Yin I.X., Zhang J., Zhao I.S., Mei M.L., Li Q., Chu C.H. (2020). The Antibacterial Mechanism of Silver Nanoparticles and Its Application in Dentistry. Int. J. Nanomed..

[65] Li F., Majd H., Weir M.D., Arola D.D., Xu H.H. (2015). Inhibition of matrix metalloproteinase activity in human dentin via novel antibacterial monomer. Dent. Mater..

[66] Zhang Y., Chen Y., Hu Y., Huang F., Xiao Y. (2018). Quaternary ammonium compounds in dental restorative materials. Dent. Mater. J..

[67] Anderson A.C., von Ohle C., Frese C., Boutin S., Bridson C., Schoilew K., Peikert S.A., Hellwig E., Pelz K., Wittmer A. (2023). The oral microbiota is a reservoir for antimicrobial resistance: Resistome and phenotypic resistance characteristics of oral biofilm in health, caries, and periodontitis. Ann. Clin. Microbiol. Antimicrob..

[68] Kreth J., Merritt J., Pfeifer C.S., Khajotia S., Ferracane J.L. (2020). Interaction Between the Oral Microbiome and Dental Composite Biomaterials: Where We Are and Where We Should Go. J. Dent. Res..

[69] Lee K.H., Wang C.Y., Tsai Y.R., Huang S.Y., Huang W.T., Kasimayan U., K.P.O. M., Chiang Y.C. (2024). Epigallocatechin gallate-immobilized antimicrobial resin with rechargeable fluorinated synergistic composite for enhanced caries control. Dent. Mater..

[70] Zhang D., Ren B., Zhang Y., Liu Y., Chen H., Xiao S., Chang Y., Yang J., Zheng J. (2020). Micro- and macroscopically structured zwitterionic polymers with ultralow fouling property. J. Colloid Interface Sci..

[71] Mangal U., Kwon J.S., Choi S.H. (2020). Bio-Interactive Zwitterionic Dental Biomaterials for Improving Biofilm Resistance: Characteristics and Applications. Int. J. Mol. Sci..

[72] Zhang N., Melo M.A.S., Bai Y., Xu H.H.K. (2014). Novel protein-repellent dental adhesive containing 2-methacryloyloxyethyl phosphorylcholine. J. Dent..

[73] Zhang N., Weir M.D., Romberg E., Bai Y., Xu H.H.K. (2015). Development of novel dental adhesive with double benefits of protein-repellent and antibacterial capabilities. Dent. Mater..

[74] Hirota K., Yumoto H., Miyamoto K., Yamamoto N., Murakami K., Hoshino Y., Matsuo T., Miyake Y. (2011). MPC-Polymer Reduces Adherence and Biofilm Formation by Oral Bacteria. J. Dent. Res..

[75] Song L., Ye Q., Ge X., Misra A., Tamerler C., Spencer P. (2017). Probing the neutralization behavior of zwitterionic monomer-containing dental adhesive. Dent. Mater..

[76] Leme-Kraus A.A., Phansalkar R.S., Dos Reis M.C., Aydin B., Sousa A.B.S., Alania Y., McAlpine J., Chen S.N., Pauli G.F., Bedran-Russo A.K. (2020). Dimeric Proanthocyanidins on the Stability of Dentin and Adhesive Biointerfaces. J. Dent. Res..

[77] Hebling J., Pashley D.H., Tjäderhane L., Tay F.R. (2005). Chlorhexidine Arrests Subclinical Degradation of Dentin Hybrid Layers In Vivo. J. Dent. Res..

[78] Lee J., Sabatini C. (2017). Glutaraldehyde collagen cross-linking stabilizes resin–dentin interfaces and reduces bond degradation. Eur. J. Oral Sci..

[79] Citta M., Anovazzi G., Basso F., Scheffel D., Zhou J., Pashley D., Souza Costa C., Hebling J. (2021). Mechanical Stability and Proteolytic Activity of Resin–dentin Bonds Using the Cross-Linked Dry Bonding Technique. Oper. Dent..

[80] Brăzdaru L., Staicu T., Albu Kaya M.G., Chelaru C., Ghica C., Cîrcu V., Leca M., Ghica M.V., Micutz M. (2022). 3D Porous Collagen Matrices—A Reservoir for In Vitro Simultaneous Release of Tannic Acid and Chlorhexidine. Pharmaceutics.

[81] Chen W., Jin H., Zhang H., Wu L., Chen G., Shao H., Wang S., He X., Zheng S., Cao C.Y. (2021). Synergistic effects of graphene quantum dots and carbodiimide in promoting resin–dentin bond durability. Dent. Mater..

[82] Firoozmand L.M., Alania Y., Bedran-Russo A.K. (2022). Development and Assessment of Bioactive Coatings for the Prevention of Recurrent Caries Around Resin Composite Restorations. Oper. Dent..

[83] Jing S.X., McDermott C.M., Flanders P.L., Reis-Havlat M., Chen S.N., Bedran-Russo A.K., McAlpine J.B., Ambrose E.A., Pauli G.F. (2024). Chemical Transformation of B- to A-type Proanthocyanidins and 3D Structural Implications. J. Nat. Prod..

[84] Reis-Havlat M., Alania Y., Zhou B., Jing S.X., McAlpine J.B., Chen S.N., Pauli G.F., Bedran-Russo A.K. (2024). Modulatory role of terminal monomeric flavan-3-ol units in the viscoelasticity of dentin. J. Biomed. Mater. Res. Part B Appl. Biomater..

[85] Al-Ammar A., Drummond J.L., Bedran-Russo A.K. (2009). The use of collagen cross-linking agents to enhance dentin bond strength. J. Biomed. Mater. Res. B Appl. Biomater..

[86] Borges L., Logan M., Weber S., Lewis S., Fang C., Correr-Sobrinho L., Pfeifer C. (2024). Multi-acrylamides improve bond stability through collagen reinforcement under physiological conditions. Dent. Mater..

[87] Carrilho M.R., Carvalho R.M., de Goes M.F., di Hipólito V., Geraldeli S., Tay F.R., Pashley D.H., Tjäderhane L. (2007). Chlorhexidine preserves dentin bond in vitro. J. Dent. Res..

[88] Gendron R., Grenier D., Sorsa T., Mayrand D. (1999). Inhibition of the activities of matrix metalloproteinases 2, 8, and 9 by chlorhexidine. Clin. Diagn. Lab. Immunol..

[89] Breschi L., Mazzoni A., Nato F., Carrilho M., Visintini E., Tjäderhane L., Ruggeri A., Tay F.R., Dorigo E.D.S., Pashley D.H. (2010). Chlorhexidine stabilizes the adhesive interface: A 2-year in vitro study. Dent. Mater..

[90] Freitas P.H., André C.B., Fronza B.M., Giannini M., Rosalen P.L., Consani S., França R. (2021). Physicochemical properties, metalloproteinases inhibition, and antibiofilm activity of doxycycline-doped dental adhesive. J. Dent..

[91] Liao S., Tang Y., Chu C., Lu W., Baligen B., Man Y., Qu Y. (2020). Application of green tea extracts epigallocatechin-3-gallate in dental materials: Recent progress and perspectives. J. Biomed. Mater. Res. Part A.

[92] Schneider-Rayman M., Steinberg D., Sionov R.V., Friedman M., Shalish M. (2021). Effect of epigallocatechin gallate on dental biofilm of Streptococcus mutans: An in vitro study. BMC Oral Health.

[93] Zhang L., Luo B., An Z., Zheng P., Liu Y., Zhao H., Zhang Z., Gao T., Cao Y., Zhang Y. (2023). MMP-Responsive Nanoparticle-Loaded, Injectable, Adhesive, Self-Healing Hydrogel Wound Dressing Based on Dynamic Covalent Bonds. Biomacromolecules.

[94] Tezvergil-Mutluay A., Agee K.A., Uchiyama T., Imazato S., Mutluay M.M., Cadenaro M., Breschi L., Nishitani Y., Tay F.R., Pashley D.H. (2011). The inhibitory effects of quaternary ammonium methacrylates on soluble and matrix-bound MMPs. J. Dent. Res..

[95] Tay F.R., Pashley D.H. (2003). Water treeing—A potential mechanism for degradation of dentin adhesives. Am. J. Dent..

[96] Moszner N., Angermann J., Zeuner F., Fischer U. Synthesis of hydrolytically stable monomers for dental adhesives. Proceedings of the IFMBE Proceedings.

[97] Gonzalez-Bonet A., Kaufman G., Yang Y., Wong C., Jackson A., Huyang G., Bowen R., Sun J. (2015). Preparation of Dental Resins Resistant to Enzymatic and Hydrolytic Degradation in Oral Environments. Biomacromolecules.

[98] Wang X., Gao G., Song H.B., Zhang X., Stansbury J.W., Bowman C.N. (2021). Evaluation of a photo-initiated copper(I)-catalyzed azide-alkyne cycloaddition polymer network with improved water stability and high mechanical performance as an ester-free dental restorative. Dent. Mater..

[99] Podgórski M., Becka E., Claudino M., Flores A., Shah P.K., Stansbury J.W., Bowman C.N. (2015). Ester-free thiol-ene dental restoratives—Part B: Composite development. Dent. Mater..

[100] Podgórski M., Becka E., Claudino M., Flores A., Shah P.K., Stansbury J.W., Bowman C.N. (2015). Ester-free thiol-ene dental restoratives—Part A: Resin development. Dent. Mater..

[101] Fugolin A.P., Pfeifer C.S. (2022). Strategies to design extrinsic stimuli-responsive dental polymers capable of autorepairing. JADA Found. Sci..

[102] Fugolin A.P., Pfeifer C.S. (2022). Engineering a new generation of thermoset self-healing polymers based on intrinsic approaches. JADA Found. Sci..

[103] Fugolin A.P.P., Ferracane J.L., Pfeifer C.S. (2022). Fatigue-Crack Propagation Behavior in Microcapsule-Containing Self-Healing Polymeric Networks. Mater. Des..

[104] Dailey M.M.C., Silvia A.W., McIntire P.J., Wilson G.O., Moore J.S., White S.R. (2014). A self-healing biomaterial based on free-radical polymerization. J. Biomed. Mater. Res. Part A.

[105] Kloxin C.J., Scott T.F., Adzima B.J., Bowman C.N. (2010). Covalent adaptable networks (CANs): A unique paradigm in cross-linked polymers. Macromolecules.

[106] Du X., Tong Y., Wang T., Zhang A., Xu Q. (2025). Self-healing and recyclable polyurethane-based solid-state polymer electrolyte via Diels-Alder dynamic network. J. Mol. Struct..

[107] Nguyen L.M.T., Truong T.T., Le H.C., Hoang M.D., Nguyen H.T., Nguyen L.T.T. (2024). Self-healing bismaleimide resin via “click” reactions: Impact of structure on healing efficiency. J. Polym. Res..

[108] Forville H., Wendlinger M., Cochinski G.D., Favoreto M.W., Wambier L.M., Chibinski A.C., Loguercio A.D., Reis A. (2025). Re-evaluating the role of moist dentin of adhesive systems used in the etch-and-rinse bonding strategy. A systematic review and meta-analysis. J. Dent..

[109] Alshaikh K.H., Hamama H.H.H., Mahmoud S.H. (2018). Effect of smear layer deproteinization on bonding of self-etch adhesives to dentin: A systematic review and meta-analysis. Restor. Dent. Endod..

[110] Ferracane J.L., Sidhu S.K., Melo M.A.S., Yeo I.-S.L., Diogenes A., Darvell B.W. (2023). Bioactive dental materials: Developing, promising, confusing. JADA Found. Sci..

[111] Braga R.R. (2021). Multifunctional restorative dental materials: Remineralization and antibacterial effect. Oral Biofilms and Modern Dental Materials: Advances Toward Bioactivity.

[112] Braga R.R., Fronza B.M. (2020). The use of bioactive particles and biomimetic analogues for increasing the longevity of resin-dentin interfaces: A literature review. Dent. Mater. J..

[113] Camilleri J. (2014). Mineral Trioxide Aggregate in Dentistry: From Preparation to Application.

[114] Wu L., Cao X., Meng Y., Huang T., Zhu C., Pei D., Weir M.D., Oates T.W., Lu Y., Xu H.H.K. (2022). Novel bioactive adhesive containing dimethylaminohexadecyl methacrylate and calcium phosphate nanoparticles to inhibit metalloproteinases and nanoleakage with three months of aging in artificial saliva. Dent. Mater..

[115] Abuljadayel R., Aljadani N., Almutairi H., Turkistani A. (2023). Effect of Antibacterial Agents on Dentin Bond Strength of Bioactive Restorative Materials. Polymers.

[116] de Castilho A.R.F., Rosalen P.L., de Souza Araújo I.J., Kitagawa I.L., Costa C., Janal M.N., Alves M.C., Duarte S., Lisboa Filho P.N., Stipp R.N. (2019). Trans, trans-farnesol, an antimicrobial natural compound, improves glass ionomer cement properties. PLoS ONE.

[117] Montoya C., Roldan L., Yu M., Valliani S., Ta C., Yang M., Orrego S. (2023). Smart dental materials for antimicrobial applications. Bioact. Mater..

[118] Darvell B.W. (2021). Bioactivity—Symphony or Cacophony? A Personal View of a Tangled Field. Prosthesis.

[119] Schmalz G., Hickel R., Price R.B., Platt J.A. (2023). Bioactivity of Dental Restorative Materials: FDI Policy Statement. Int. Dent. J..

[120] Moussa D.G., Fok A., Aparicio C. (2019). Hydrophobic and antimicrobial dentin: A peptide-based 2-tier protective system for dental resin composite restorations. Acta Biomater..

[121] de Sousa J.P., Carvalho R.G., Barbosa-Martins L.F., Torquato R.J.S., Mugnol K.C.U., Nascimento F.D., Tersariol I.L.S., Puppin-Rontani R.M. (2019). The Self-Assembling Peptide P(11)-4 Prevents Collagen Proteolysis in Dentin. J. Dent. Res..

[122] Nakabayashi N., Kojima K., Masuhara E. (1982). The promotion of adhesion by the infiltration of monomers into tooth substrates. J. Biomed. Mater. Res..

[123] Van Meerbeek B., Conn L.J., Duke E.S., Eick J.D., Robinson S.J., Guerrero D. (1996). Correlative transmission electron microscopy examination of nondemineralized and demineralized resin-dentin interfaces formed by two dentin adhesive systems. J. Dent. Res..

[124] Mazzoni A., Nascimento F.D., Carrilho M., Tersariol I., Papa V., Tjäderhane L., Di Lenarda R., Tay F.R., Pashley D.H., Breschi L. (2012). MMP activity in the hybrid layer detected with in situ zymography. J. Dent. Res..

[125] Zhang A., Ye N., Aregawi W., Zhang L., Salah M., VanHeel B., Chew H.P., Fok A.S.L. (2021). A Review of Mechano-Biochemical Models for Testing Composite Restorations. J. Dent. Res..

[126] Culp D.J., Quivey R.Q., Bowen W.H., Fallon M.A., Pearson S.K., Faustoferri R. (2005). A mouse caries model and evaluation of aqp5-/- knockout mice. Caries Res..

[127] Wen Z.T., Huang X., Ellepola K., Liao S., Li Y. (2022). Lactobacilli and human dental caries: More than mechanical retention. Microbiology.

